# “In the Same Storm, but Not on the Same Boat”: Children Grief During the COVID-19 Pandemic

**DOI:** 10.3389/fpsyt.2021.638866

**Published:** 2021-01-26

**Authors:** Sara Albuquerque, Ana R. Santos

**Affiliations:** ^1^Center for Research in Neuropsychology and Cognitive and Behavioral Intervention, Faculty of Psychology and Educational Sciences, University of Coimbra, Coimbra, Portugal; ^2^Clinical, Research and Training Center “PIN - em todas as fases da vida”, Lisbon, Portugal; ^3^Universidade Lusófona, HEI-Lab, Lisbon, Portugal; ^4^Department of Pediatric Psychiatry – Hospital Fernando Fonseca, Lisbon, Portugal

**Keywords:** COVID-19, grief, death, children, childhood, bereavement

## Introduction

COVID-19 has profoundly shaken the world and changed the lives of children and families. People around the world are mourning a sense of freedom, normalcy, and routine. However, although in the same “storm,” children are not on the same boat as adults. Children may be at a higher risk for mental health effects, given their limited capacity to understand their surroundings, cope with stressors, and control their environments. In fact, research already demonstrates that COVID-19-related rates of depression and anxiety are prevalent among children and adolescents (1).

Research has shown that victims of the virus will likely leave behind a large number of grieving children and grandchildren, with rates of 2.2 children and 4.1 grandchildren bereaved for each person who dies (2). There is evidence on the failure of addressing the needs of bereaved children proactively and on an ongoing basis (3), which may result in poor mental and physical health (4). Also, it is estimated that 5 to 10% of children and adolescents who suffer the loss of a loved one develop clinically significant psychiatric difficulties (5); this number may be higher given the specificities of loss in the context of COVID-19. Finally, the evidence on childhood trauma and loss as a risk factor for adult psychopathology (6), highlights the need for early identification and intervention.

The development of specific knowledge about children grief under the COVID-19 circumstance is therefore urgent. This opinion paper aimed at highlighting the challenges and needs of grieving children.

## Children's Particular Vulnerability

Children may experience various stressors related to COVID-19, such as disruptions to their routines, worrying about friendships changing, not being able to catch up academically, while also grieving the loss of childhood rites of passage, such as school ceremonies and events or organized sports. Other factors pertain to the experience of an intensive fear of themselves or a loved one contracting the virus and exposure to disturbing media content or distressing adult conversations. Also, children might be more distant from those who might support them—friends, teachers, wider family–and some, especially those which parents are essential workers or first responders, might be physically distant from their caregivers. Similarly, economic instability, job loss, or disrupted access to healthcare or other support services might be present. All of this can increase family stress and conflict, abuse and domestic violence (7). If, in the context of these already challenging stressors a death in the family occurs, the impact is undoubtedly paramount.

The particular circumstances associated with COVID-19 deaths may constitute a significant risk factor for children's grief process, as studies have shown that unexpected and sudden losses or contexts of multiples deaths in the family are particularly impactful, even in the absence of previous psychological vulnerability (8). In the current context, people may sicken and die quite swiftly and a sense of unpredictability and shock may prevail, even if the person that died had already a frail physical health. Also, children won't be able to spend time with their dying relative or possibly attend the funeral due to sanitary restrictions.

In addition, children may be dealing with this for the first time, and therefore have less knowledge on life and death issues. They are more prone to misinterpret the available information and, personally, have no roadmap regarding expected feelings, reactions and possible coping strategies. Also, the tendency that younger children have to live more in the present, with moments of expression of suffering being more transitory, might contribute to underestimate the impact of the loss (9).

Other aspects that may contribute to this underestimation are the misperception that the loss was somewhat expected (e.g., due to the advance age or pre-existing illness of the deceased), the social stigma against the COVID-19 patients and their families (10) and the dehumanization of death as it is simply presented as statistics.

## Emotional Regulation Challenges in Grieving Children

Children are observed to express their sadness and anger in a sporadic manner, interspersed with long periods of normal behavior and activity such as play (11). These transitions between bursts of sadness and normal behavior resemble the concept of oscillation in the Dual Process Model (12), defined as a back and forth in focusing feelings of grief and loss (Loss coping orientation–LO) and coping with everyday life stressors and taking a “time off” from grieving (Restoration coping orientation–RO).

The circumstances of death and dying in the current context may pose particular challenges to this important self-regulatory strategy. Firstly, children are in nature more restorative but access to activities (e.g., play dates with friends, sports, extra-curricular activities) that might contribute to respite from the sadness and loss, are currently more limited. On the other hand, the death-related cues (masks, stories of people affected by COVID, media exposure) may compromise RO coping as children are constantly being reminded of the death (7).

To make this context even more complex, not only has the child suffered a significant loss, but the person they would usually turn to—their parents–may be too distressed to provide adequate support. Parents may be experiencing an overload of RO stressors (13), as some may be faced with job or financial uncertainties; and juggling multiple roles and needs at the same time (e.g., working from home and having to care for the children at the same time). Parents may also be grieving a significant loss themselves (e.g., loss of partner or loss of their mother or father). Children rely deeply on the functioning of the parent, who is vital in modeling, supporting and giving permission for the child's grieving (14). The perception of vulnerability in the parents and the will to protect them may stop children from talking about their reactions (15), which may compromise the focus in LO coping strategies. Similarly, missing final moments with the loved one, not having the chance to say goodbye and participating in the funeral may contribute to feelings that the person's death doesn't feel real and that something is unfinished or unresolved (16).

## Challenges in Identifying Children at Greater Risk

Early and accurate identification of mental health problems is key to early intervention. However, identifying children's maladaptive grief is challenging. Firstly, there may be symptoms and behaviors with significant impact but that they do not recognize as being related to the loss (9). Typically, these reactions take the form of physical symptoms or maladaptive behaviors that can mimic other mental health issues; for example, it can manifest as aggressive or oppositional behavior and be mislabeled with oppositional defiant disorder.

Also, predictions of delayed grief—suppressed, postponed or inhibited grief responses—have been proposed (17) and this may be a particular risk for children. A mediating factor in this type of reaction is the lack of social support at the time of loss (9), which may be exacerbated due to current social restrictions and depletion of emotional resources in the caregivers (e.g., parents).

Finally, loss in childhood should be examined in the context of a child's cognitive, emotional, and social development (18). As children's development is ongoing, so is their grief. Feelings of loss are often continually processed as children gain more understanding of their loss (19). Therefore, children grief can manifest in different ways throughout their development and lifespan, adding complexity to its adequate evaluation.

## The Value of Information and Communication

Research had shown that children want to receive more information about the death from their parents as they went through the grieving process (20). Having access to information, with the due cautions, in the context of the pandemic, is especially important as the scenarios are often unpredictable and constantly changing. Therefore, it is important to reflect on the potential obstacles to this communication. Adults may believe that by not speaking, they are protecting the child, as if this protection relieved the pain and changed the reality of the loss (16, 21). Also, it might be helpful for adults to gain awareness of their patterns of coping strategies with their own suffering and death and how these may impede connecting and communicating with the child. Children of all ages often know more about what is happening than adults realize (21); they might overhear conversations or phone calls; they can be given information—not always accurate—by others; and very often they intuitively pick up on changes in their family's mood and emotions. Children depend on their parents or significant adults to acquire their information about the world, to learn about what to do with their emotional pain and to access the truth. However, balancing truth with reassurance is critical (9). In the current context it is important to provide information about the virus, its different effects on the body and stages of the loved one's illness, and on the death, always in line with the needs and questions of the child.

Also, school can serve as a safe haven, especially under the current circumstance as parental support might be less available. Schools should therefore have information on grief symptoms and impact on school behavior. Also, the needs of bereaved children should be addressed proactively and on an ongoing basis, with respect for autonomy and freedom in students' emotional expression (16). In addition, school-based interventions focusing on improving children's well-being are important. A systematic review highlighted promising results in this area of school-based randomized controlled trials focusing on enhancing youth's emotional and social skills (22). Examples of recognizable features of positive youth development consist of bonding, social, emotional, cognitive, behavioral, and moral competence; resilience, self-determination, spirituality, self-efficacy, clear and positive identity, belief in the future, prosocial norms (healthy standards for behavior); recognition of positive behavior and opportunities for prosocial involvement (23). With grieving children, it is important to also acknowledge a more positive view of mental health.

## Conclusion

When facing grief in the context of COVID-19, children and adults are not in the same boat, as they use different ways to sailing, stop or survive. The recognition of these differences is essential to adequately attend to the child's needs. Nevertheless, grieving in the current context can be a great equalizer between children and adults. The COVID-19 pandemic has altered the perception of security and predictability essential for all when looking at the world and relationships. Both adults and children experience changes in their routine, social limitations and the pain of not saying goodbye. Enhanced empathy and connectedness through this shared experience is possible but only if the culture of silence surrounding death is not perpetuated. In this opinion paper we aimed at increasing awareness for grieving children needs and challenges in the current context in the hope that it may inform not only intervention but also prevention strategies of bereavement complexities.

## Author Contributions

SA and AS contributed to the development of the idea and the literature research and the writing of the manuscript. All authors contributed to the article and approved the submitted version.

## Conflict of Interest

The authors declare that the research was conducted in the absence of any commercial or financial relationships that could be construed as a potential conflict of interest.
